# Mediastinal mayhem: the challenges of incidental thoracic arteriovenous malformations—a case report

**DOI:** 10.1093/ehjcr/ytae454

**Published:** 2024-08-27

**Authors:** Louise Tram, Phillip Freeman, Robert Johannes Leusink

**Affiliations:** Department of Radiology, Aalborg University Hospital, 9000 Aalborg, Denmark; Department of Cardiology, Aalborg University Hospital, Aalborg, Denmark; Department of Radiology, Aalborg University Hospital, 9000 Aalborg, Denmark

**Keywords:** Case report, Arteriovenous malformation, Vascular anomalies, Aortic valve replacement, Coronary angiography, Bicuspid aortic valve

## Abstract

**Background:**

Arteriovenous malformations (AVMs) within the mediastinum are rare vascular anomalies. With the increasing number of coronary angiographies being performed, the number of incidentally found cases is rising. This presents challenges in terms of determining the appropriate treatment strategy.

**Case summary:**

We present the case of a 79-year-old man with aortic stenosis, experiencing functional dyspnoea and fatigue. Echocardiography showed a bicuspid aortic valve, and while left heart catheterization confirmed no significant coronary stenosis, it revealed a tortuous vessel originating from the circumflex artery (Cx), assumed to be heading toward the pulmonary circulation. The patient was scheduled for a surgical replacement of the aortic valve (SAVR). During the SAVR, the tortuous vessel was revealed to be a large, complex AVM located in the mediastinum. This increased both the duration of the surgery and the use of cardioplegia. Further, bleeding occurred per-operatively. Post-operatively, the patient developed tachy-brady syndrome and was treated with a pacemaker before discharge.

**Discussion:**

Due to the rarity of incidental AVMs in the middle/posterior mediastinum, no standard treatment protocol is available. This leaves clinicians and surgeons to manage the disease on a case-by-case basis, often with limited experience to guide their decisions. This patient case underscores the challenge of determining whether patients should be offered transcatheter aortic valve implantation (TAVI) or surgery. Furthermore, it highlights the intricate challenges that can arise when dealing with thoracic AVMs during cardiac procedures, emphasizing the importance of pre-operative awareness and tailored surgical approaches based on multidisciplinary discussions.

**Learning points**

- In this case, the presence of the thoracic arteriovenous malformations (AVMs) was not initially recognized but impacted the surgical procedure.
- The post-operative course can be complex in cases involving thoracic AVMs, and complications may arise. This underscores the need for careful evaluation and planning to optimize patient outcomes.

## Introduction

Arteriovenous malformations (AVMs) within the mediastinum are rare vascular anomalies that present challenges in terms of determining appropriate treatment strategies and understanding their clinical presentation.^1^ Often, these AVMs are discovered incidentally in asymptomatic patients during unrelated medical procedures such as coronary angiography or coronary computed tomography (CT).

We present a case involving a patient with an untreated AVM located in the mediastinum, which had not been recognized prior to undergoing surgical aortic valve replacement (SAVR). This case highlights the challenges that can arise when dealing with thoracic AVMs during cardiac procedures, underscoring the importance of pre-operative awareness, and the need for tailored surgical approaches informed by multidisciplinary discussions.

## Summary figure

**Figure:**
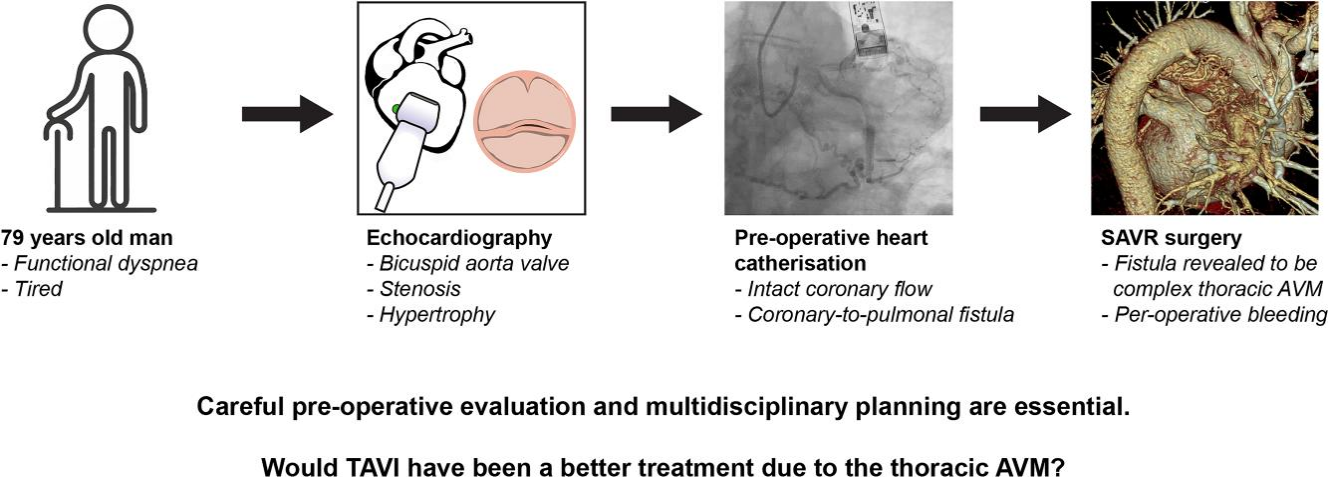


## Case presentation

The cardiology outpatient clinic received a referral for a 79-year-old man diagnosed with aortic stenosis. He was experiencing dyspnoea with activity and fatigue, but otherwise maintained a high level of functional activity. During the first check-up at the clinic, it was observed that the patient appeared well-compensated. Upon auscultation of the heart, a grade III–IV ejection murmur was detected at the base, along with a partially diminished second heart sound. Echocardiography revealed hypertrophy of the left ventricle and calcification of the aortic valve, with a bicuspid aortic valve (BAV). The mean gradient was measured at 38 mmHg, and the aortic valve area was estimated to be between 0.8 and 1.0 cm^2^ with no indication of aortic insufficiency. Additionally, the echocardiography revealed calcification of the mitral valve annulus and a mild mitral stenosis, with a mean gradient of 6 mmHg.

**Figure 1:**
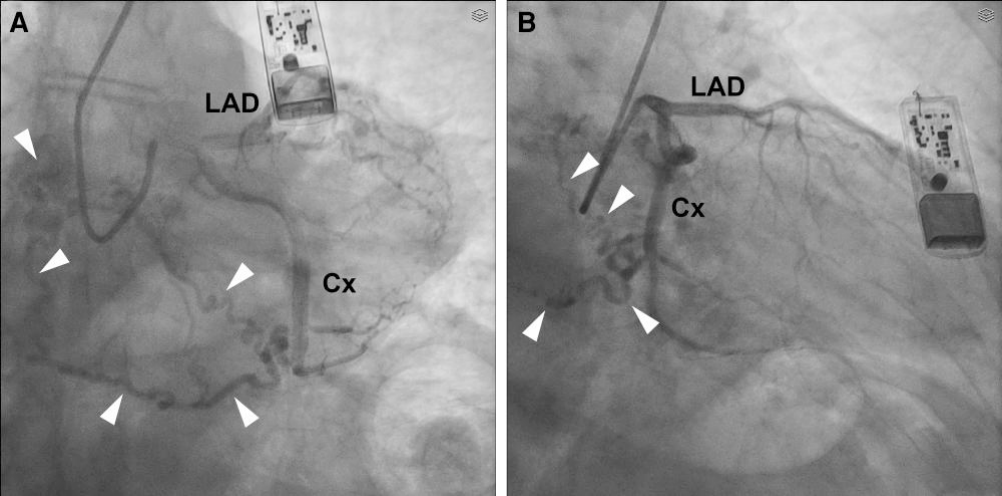
Coronary angiography showing a vessel that appeared to connect the circumflex artery to the pulmonary circulation. *(A)* RAO 60° projection. *(B)* RAO 30° projection. White arrowheads mark the arteriovenous malformations. LAD, left anterior descending artery; Cx, circumflex artery; AVM, arteriovenous malformation.

As a result, the patient was scheduled for left heart catheterization to assess coronary blood flow. This procedure confirmed the absence of coronary stenosis. However, it also revealed a tortuous vessel originating from the circumflex artery (Cx), which was presumed to lead to the pulmonary circulation (*Figure 1*). Initially, this blood vessel was diagnosed as a-coronary-to pulmonary fistula (CPF).^2^ Subsequent investigations showed that the patient had a normal pulmonary pressure, around 22 mmHg, and an arterial blood gas analysis did not suggest shunting.

**Figure 2:**
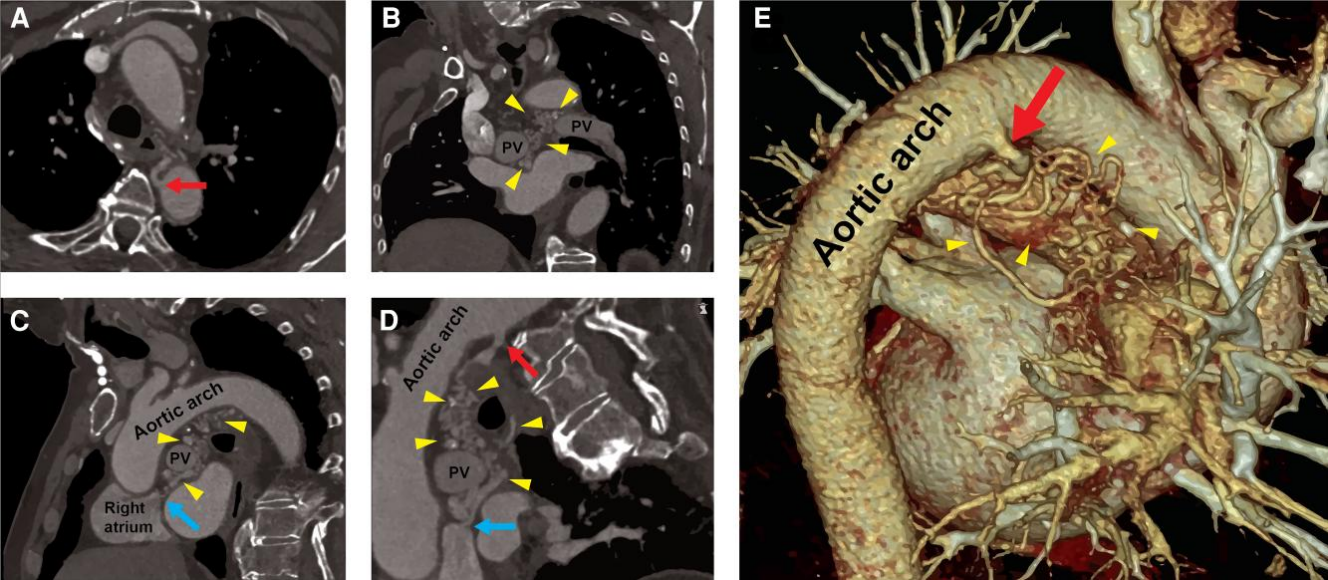
Computed tomography showing an overview of the mediastinum, revealing that the vessel visualized in the angiography was not a simple arteriovenous fistula but rather an arteriovenous malformation. *(A)* Axial view. *(B)* Coronal view. *(C)* Sagittal view. *(D)* A vessel tracking reconstruction of the arteriovenous malformation. *(E)* 3D reconstruction of the mediastinal arteriovenous malformation. Red arrow marks the origin of the arteriovenous malformation from the left bronchial artery. Blue arrow marks connection of the arteriovenous malformation to the origin of the right atrium. Yellow arrowheads mark the arteriovenous malformation. PV, pulmonary vein; AVM, arteriovenous malformation; CT, computed tomography.

Considering these findings and symptoms, the patient was referred for aortic valve replacement. Prior to surgery, a pre-operative transoesophageal echocardiography and a transcatheter aortic valve implantation-computerized tomography (TAVI-CT) were performed. The TAVI-CT unexpectedly revealed that what was previously diagnosed as a CPF was actually a complex AVM located in the posterior mediastinum. This AVM originated from the left bronchial artery, connected to the coronary arteries, and drained to the distal superior vena cava at the origin of the right atrium (*Figure 2*).

Following this, the patient was scheduled for a replacement of the aortic valve, opting for a biological valve replacement. The SAVR was performed via median sternotomy. The valve replacement was successful, but the operating time was prolonged because of re-bleeding from the left main coronary artery. The bleeding was caused by the AVM connecting to the Cx and necessitated an increased use of cardioplegia during the perioperative period. During hospitalization, the patient developed tachy-brady syndrome and was treated with a pacemaker before discharge. A follow-up 4 months after the operation showed a well-functioning aortic valve.

## Discussion

So far, only a few cases of AVM in the middle/posterior mediastinum have been reported, all of which have been symptomatic and thus necessitated treatment.^3,4^

Due to the rarity of incidental AVM in the middle/posterior mediastinum, no standard evidence based optimal protocolized treatment is available, which leaves clinicians and surgeons to treat these anatomical variants on a case-by-case basis with little experience to base decisions on.

The treatment strategy is based on the patient’s general condition and the observed anatomical variations, guiding the choice between TAVI and SAVR.^5^ The two treatments have a similar risk profile and outcome.^6^ According to the European guidelines, the age of the patient usually leans towards TAVI instead of SAVR.^7^ However, due to the presence of a BAV, the patient was offered a SAVR.^8^ In this particular situation, the AVM was not clearly recognized during the heart team conference, and the importance of this finding—especially the potential issues with cardioplegia—was not fully appreciated. As a result, it did not influence the planning of the surgery or the decision to proceed with TAVI instead of SAVR.

One could argue that even with the BAV diagnosis, TAVI might have been preferable due to its potential to instantly visualise the impact on the flow in the AVM and the coronary arteries, especially in the Cx. Choosing TAVI could have avoided the complications related to cardioplegia and intraoperative bleeding encountered during SAVR. Furthermore, TAVI would have provided the possibility to coil the AVM/left bronchial artery immediately during the procedure. Immediate coiling of the AVM might have reduced the duration of the surgery time and minimized the risk of the patient developing tachy-brady syndrome post-operatively.^9^

The rarity and complexity of thoracic AVMs, as seen in this patient case, often lead to uncertainty in selecting the most appropriate approach. This uncertainty can result in less than ideal outcomes or necessitate multiple interventions. In this patient case, the presence of a thoracic AVM complicated the surgical procedure, and the standard approach with SAVR for aortic valve replacement for patient with BAV limited the perioperative opportunities for treating the AVM. This case report aims to highlight the complexities inherent in managing thoracic AVMs, emphasizing the importance of a comprehensive evaluation and planning prior to surgery to optimize patient outcomes. A multidisciplinary approach, involving cardiologists, cardiothoracic surgeons, radiologists, and radiologists, is crucial in ensuring thorough assessment and coordinated care, which can significantly improve surgical outcomes and reduce the risk of complications.

In the end, the decision to intervene or not to intervene can still only be made on a case-by-case basis, until standardized guidelines on the management of incidental thoracic AVMs are established.

## Lead author biography


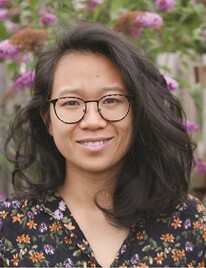


Louise Tram is a resident physician in radiology at Aalborg University Hospital in Denmark. Her primary interest lies in the diagnostic field, with a particular focus on endovascular repair. She intends to pursue further studies in intervention.

**Consent:** The authors confirm that they have obtained written consent for the submission and publication of this case report, including images, in accordance with COPE guidelines.

**Funding:** None declared.

## Data Availability

No new data were generated or analysed in support of this research.
